# Association of Interleukin-1B gene Polymorphism with *H. pylori* infected Dyspeptic Gastric Diseases and Healthy Population

**DOI:** 10.12669/pjms.36.4.1883

**Published:** 2020

**Authors:** Furkhanda Kalsoom, Sajjad-ur-Rahman, Muhammad Shahid Mahmood, Tahir Zahoor

**Affiliations:** 1 Dr. Furkhanda Kalsoom, Institute of Microbiology, University of Agriculture, Faisalabad, Pakistan; 2 Prof. Dr. Sajjad-ur-Rahman, Post Doc, Director, Institute of Microbiology, University of Agriculture, Faisalabad, Pakistan; 3 Dr. Muhammad Shahid Mahmood, Ph.D. Associate Professor, Institute of Microbiology, University of Agriculture, Faisalabad, Pakistan; 4 Prof. Dr. Tahir Zahoor, Post Doc, Director, General National Institute of Food Sciences and Technology, University of Agriculture, Faisalabad, Pakistan

**Keywords:** Cytotoxic antigen gene A (Cag A), Dyspepsia, *H. pylori*, Gastritis, Peptic ulcer

## Abstract

**Objective:**

The aim of study was to investigate the association of IL 1B gene polymorphism with involvement of *H. pylori* and other gastric diseases.

**Methods:**

Blood samples of dyspeptic patients were collected from endoscopy department of Allied Hospital Faisalabad from January 2017 to January 2019 and were qualitatively assayed for serological detection of CagA *H. pylori* antibodies. PCR followed by direct sequencing was performed for proinflammatory IL-1B gene polymorphism detection. Sequence analysis was performed in software SnapGene viewer for haplotypes.

**Results:**

Demographic characteristics of seropositive patients showed maximum 25% gastritis in age groups of 20-40 years and 41-60 years, predominantly (41.7%) in females. While in seronegative patient’s gastritis (33.3%) was found in age group of 20-40 years mainly in males (41.7%). Among studied groups, higher expression of IL-1B-511 genotype (33.3%) polymorphism was found in healthy individuals as compared to *H. pylori* seropositive (25%) and seronegative (8.3%). While IL-1B-31 genotype showed maximum 33.3% polymorphism rate in seropositive gastric diseased group. Moreover, haplotypes frequencies IL-1B-511CC and IL-1B-31TT were predominantly (20%) found in seropositive gastric diseased group.

**Conclusions:**

In *H. pylori* seropositive patients, gastric disease was commonly found, however, gastric disease was not only associated with *H. pylori* as seronegative patients were also carrying gastric complications. Interleukin IL-1B polymorphism was partially associated with *H. pylori* infection in studied dyspeptic population.

## INTRODUCTION

*Helicobacter pylori* (*H. pylori*) is a Gram-negative rod shape bacterium that colonizes stomach of about 50% of world’s population.^1^ It can colonize the gastric mucosal environment for years if left untreated and increase the risk of gastric disease development.^2^ Frequent physiological changes by this bacterium are gastritis, peptic ulcer (PU) and less commonly gastric cancer.^3^ *H. pylori* has been classified as class I carcinogen by the World Health Organization.^4^ *H. pylori* is considered as a major cause of peptic ulcer and mucosa associated lymphoid tissue lymphoma or gastric cancer.^5^ Although, gastritis to gastric cancer development is a rare state, various studies reported that IL-1 and tumor necrosis factor- alpha (TNF-α) polymorphism along with *H. pylori* infection are predisposing risk factors for gastric carcinoma.^6^ *H. pylori* infection induces proinflammatory host response in stomach and leads to release of different cytokines or interleukins, more frequently IL-1B, IL-1A, IL-6, IL-8, IL-10, and TNF-α.^7^,^8^ Interleukin polymorphism increases the production of mucosal cytokine (IL-1β) level that ultimately reduces the acid (HCl) secretion in the stomach and causes gastric inflammation.^9^

Various studies have demonstrated that expression of IL-1B gene is frequently influenced by two allelic variants, IL-1B-511 and IL-1B-31, which are associated with IL-1B transcription.^10^ The polymorphism of these two genotypes has a synergistic effect on phenotypic change that increases the production of cytokine level and results in predisposition of gastritis.^11^ Several studies have reported that chronic gastritis is an established precursor of gastric adenocarcinoma with involvement of cytokine gene polymorphism.^12^ A study described the association of genotypes (IL-1 or 1L-8) polymorphism and *H. pylori* infection and reported their combined effect for the risk of gastric carcinogenesis.^13^ In addition, haplotypes (TT, CC or CT) variable frequencies have an association with gastritis and gastric cancer development.^14^ The aim of our study was to elucidate the potential association of *H. pylori* infection with IL-1B gene polymorphism existence in infected population in district Faisalabad, Pakistan.

## METHODS

### Study subjects

A total of 240 dyspeptic patients were examined through endoscopy for presence of gastric disease at tertiary care hospital, Allied Faisalabad. This study was conducted from January 2017 to January 2019. A structured questionnaire was designed to collect demographic data of enrolled patients. Age of participants was categorized in three groups; 20-40 years, 41-60 years and 61-90 years including both (male and female) genders. The patients unable to complete the endoscopy procedure were excluded due to failure of clinical indication and informed consent was signed by patient or patient`s attendant for blood sample collection. Prior to conduct study approval was obtained from local health committee Allied Hospital Faisalabad (D.No.194/ORIC dated January 1, 2019). The bioethics committee UAF also approved the study protocol. A group of healthy volunteer individuals was also enrolled to compare interleukin (IL-1B) gene polymorphism among clinically gastric diseased and healthy population at district Faisalabad.

### H. pylori Serological Examination

All selected dyspeptic patients were initially screened for presence of *H. pylori* infection on the basis of antibodies (Ab/s) detection. All blood samples were processed on *H. pylori* ’One Step Test device (CTK BIOTECH, San Diego, CA 92121 USA).^15^

### DNA Extraction

A total of 36 samples, 12 from each selected group (Healthy, *H. pylori* seropositive and seronegative gastric diseased) were processed for IL-1B gene polymorphism as only *H. pylori* seropositive were entertained with comparison of others. Host genomic DNA extraction was performed by using commercially available kit (HiPura Blood Genomic DNA Kit) as described previously,^16^ and extracted DNA samples were stored at 4ºC for genotyping.

### Genotyping for IL-1B gene Polymorphism

All extracted DNA samples were processed further for IL-1B genotyping and amplification was done through PCR using commercially available PCR kit (Thermo Scientific™ K0171). Polymorphism frequency was analyzed by processing the PCR products on 2% gel electrophoresis, visualizing a single band of specific base pair sizes in patients and healthy group samples. Specific primer sets and PCR conditions were used as followed in a previous study.^9^ The sequences of reverse and forward primers and PCR conditions are given in (Table-I).

**Table-I:** PCR primers and conditions.

Genotype polymorphism	Primers	PCR conditions
IL-1B-511C/T	F 5′-GC CT GA AC CC TG CA TA CC GT-3′ R 5′-GG AA TC TT CC CA CT TA CA GA TGG-3′	Denaturation of DNA for 10 minutes at 95°C, 40 cycles for 1 minute at 95°C, annealing at 55°C extension at 72°C and final extension for 5 minute at 72°C.
IL-1B-31T/C	F 5′-AG AA GC TT CC ACCAATAC TC-3′ R 5′-AC TAAC TT TA GG GT GT CAG-3′	10 min denaturation at 94°C, 36 cycles for 2 minute at 94°C, annealing at 54°C, extension at 72°C and final extension at 72°C for 5 min.

For allelic variant analysis (IL-1B-511 C/T and IL-1B-31 T/C allele haplotypes), PCR products were sent to Eurofins Genomics, USA. Sequencing results were annotated in software SnapGene (version 4.2.1) for evaluation of allelic haplotype (CC, TC and TT) frequencies.

## RESULTS

### Segregation of Selected Dyspeptic Population

**Fig.1:**
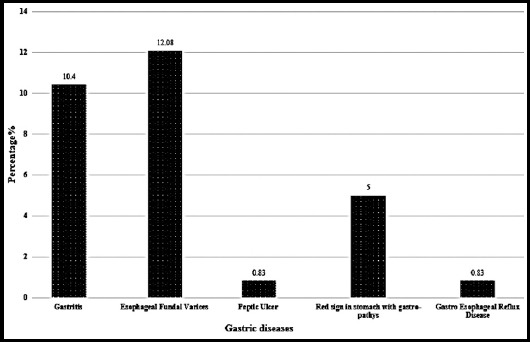
Clinical characteristics of dyspeptic gastric diseased patients.

Out of 240 dyspeptic patients, 70 (29.8%) were diagnosed with gastric diseases, of which 25 were having gastritis, 29 were with esophageal fundal varices (EFV), 12 showed red sign in stomach with gastro-pathy while only 2 patients were having peptic ulcer and 2 patients showed gastro esophageal reflux disease (GERD). The categorical distribution of dyspeptic gastric diseased patients is shown in (Fig.1).

Demographic characteristic of gastric diseased patients showed that 50% of population were found in second age group, 28.6% in first while only 21.4% were found in third age group. Gender wise distribution showed 55.7% were females.

### Seroprevalence of H. pylori

In overall selected dyspeptic population, a low seroprevalence (7.0%) of *H. pylori* was found, while among 70 gastric diseased patients, 16 (23%) were positive for CagA *H. pylori* antibodies and others 54 (77%) were seronegative. Most of the seropositive gastric diseased patients (43.8%) were found in the first age group and predominantly were females (56.2%).

### Characteristics of selected clinical and healthy groups

Studied subjects (n=36) were found with variable clinical characteristics. Table-I. Maximum gastritis 33.3% cases of seronegative gastric diseased group were found in the first age group. While, 25% of gastritis cases were found in other two age groups of seropositive and seronegative gastric diseased cases respectively. In healthy group, maximum 41.7% individuals were found in first and second age group. Gender wise distribution showed that majority of the cases (58.3%) were females in seronegative and healthy groups. EFV cases were found maximum 16.7% in first and second age groups of seropositive and seronegative gastric diseased patient respectively. PU occurrence rate of 8.3% was found in first age group of seronegative while in other two age groups of seropositive patients. Demographic distribution of three groups selected for genotype IL-1B polymorphism is shown in (Table-II).

**Table-II:** Demographic distribution of clinical and healthy individuals for IL-1B Genotype.

	Healthy group No. of Subjects (%)	H. pyloriSeronegative Gastric diseased group No. of cases (%)			H. pylori Seropositive Gastric diseased group No. of cases (%)		

Parameters		Gastritis	PU*	EFV**	Gastritis	PU*	EFV**
***Age***							
20-40 Years	5 (41.7)	4 (33.3)	1 (8.3)	-	3 (25)	-	2 (16.7)
41-60 Years	5 (41.7)	3 (25)	-	2 (16.7)	3 (25)	1 (8.3)	-
61-90 Years	2 (16.7)	1 (8.3)	-	1 (8.3)	2 (16.7)	1 (8.3)	-
***Gender***							
Male	5 (41.7)	5 (41.7)	-	-	3 (25)	1 (8.3)	2 (16.7)
Female	7 (58.3)	3 (25)	1 (8.3)	3 (25)	5 (41.7)	1 (8.3)	-

* Peptic Ulcer (PU),

** Esophageal Fundal Varices (EFV).

### Genotype and Allelic variant distribution of IL-1B polymorphism in clinical and healthy Individuals

Out of 36 cases, 23 showed IL-1B gene polymorphism on targeted positions (-511 and -31). Maximum 25% polymorphism was found at IL-1B-511CT in *H. pylori* seropositive gastric diseased cases, while for IL-1B-31TC genotype polymorphism existence was high 16.7% in both gastric diseased cases. Twenty-five percent of gastritis patients showed polymorphism for both (IL-1B-511 and IL-1B-31) genotypes whereas, only 8.3% of EFV patients showed polymorphism for IL-1B-31T/C. Healthy individuals also showed 33.3% and 16.7% polymorphism at IL-1B-511C/T and IL-1B-31T/C promoter sites respectively. The detail of IL-1B gene polymorphism is presented in (Table-III). All samples positive for IL-1B-511 T/C genotype polymorphism, produced a single band of 155bp size amplicon while IL-1B-31 C/T positive samples exhibited a single band of 448bp in PCR products as visualized on 2% gel electrophoresis.

**Table-III:** Comparative genotype frequencies in relation IL-1B gene polymorphism in seropositive gastric diseased patient and healthy group.

Genotype	Seronegative Healthy group No. of individuals (%)	Seronegative Gastric Diseased group No. of cases (%)			Seronegative Gastric Diseased group No. of cases (%)		

		Gastritis	PU	EFV	Gastritis	PU	EFV
IL-1B-511C/T	4 (33.3)	1 (8.3)	-	-	-3 (25)	-	-
IL-1B-31T/C	2 (16.7)	2 (16.7)	-	1 (8.3)	-2 (16.7)	-1 (8.3)	-1
IL-1B-511C/T plus IL-1B-31 T/C	-	3 (25)	-	-	-3 (25)	- -	-(8.3)
Negative	6 (50)	2 (16.7)	1 (8.3)	2 (16.7)	1 (8.3)	-1 (8.3)	-

*Peptic Ulcer (PU), **Esophageal Fundal Varices (EFV).

### Haplotype C-T allelic variants in clinical and healthy groups

Two loci in IL-1B gene (IL-1B-511C/T and IL-1B-31T/C) containing three main haplotypes (CC, CT and TT) were analyzed in the studied population. Out of 23 individuals (clinical and healthy) with polymorphic IL-1B genotype, the persistence of polymorphism was compared between seronegative and seropositive groups. For IL-1B-511, seronegative healthy individuals showed maximum 33.3% frequency of CT haplotype, whereas, high 20% of CC haplotype carriers were found in seropositive gastric diseased patients. While, 14.3% CT haplotype frequency was found for IL-1B-511 in gastric diseased seronegative patients. Individuals carrying IL-1B-31 genotype were found to have maximum 28.6% haplotype CC in seronegative gastric diseased cases as it was 20% among seropositive gastric diseased cases. Similarly, the frequency of haplotype TT was also prevalent in 20% cases of seropositive gastric disease and CT allelic variant was 14.3% among seronegative gastric diseased cases. The polymorphism of both genotypes; IL-1B-511 and IL-1B-31 was simultaneously identified among seronegative gastric diseased cases with 28.6% TT haplotype frequency. While, 20% CC haplotype was found in seropositive cases followed and 10% CT haplotype. Seronegative gastric diseased cases (14.3%) were found with CC haplotype. Haplotype distribution in clinical cases and healthy individuals was mentioned in (Table-IV).

**Table-IV:** Genotype and haplotype allelic distribution of IL-1B polymorphism in clinical patients with gastric disease and healthy individuals.

Genotype	Haplotype	Healthy individuals (%)	Seronegative Gastric Diseased Cases (%)	Seropositive Gastric Diseased Cases (%)
IL-1B-511	CC	1 (16.7)	-	2 (20)
	CT	2 (33.3)	1 (14.3)	-
	TT	1 (16.7)	-	1 (10)
	CC	-	2 (28.6)	2 (20)
IL-1B-31	CT	1 (16.7)	1 (14.3)	-
	TT	1 (16.7)	-	2 (20)
	CC	-	1 (14.3)	2 (20)
	CT	-	-	1 (10)
IL-1B 511plus IL-1B-31	TT	-	2 (28.6)	-

## DISCUSSION

In the present study out of total dyspeptic population, 29.2% were found with gastric diseases. The prevalence of EFV was found higher 12.08% as compared to gastritis (10.4%) and PU (0.83%) irrespective of gender and age. *H. pylori* seroprevalence in gastric diseased patients was found as 23%. The present study showed an independent correlation between gastric diseases and *H. pylori* infection as gastric atrophy was present in both groups dyspeptic population (with or without *H. pylori* infection). These results indicated that *H. pylori* is not the only cause of gastric abnormalities. In consistency with a previous study that host genetic factors and unhygienic living condition are involved in diverse effects of *H. pylori*-related gastric diseases.^17^

The role of genetic risk factor was also investigated for development of gastric disease severity. The results of the study showed that seropositive gastric diseased patients with gastritis had higher rate 25% of IL-1B gene polymorphism at -511C/T site as compared to seronegative gastric diseased group with gastritis (8.3%). The IL-1B gene polymorphism at -31T/C site was also studied and it was found that all the groups (seronegative, seropositive and healthy population) expressed same rate (16.7%). There were some of the patients with gastritis showed polymorphism for this gene at both of these sites (IL-1B-511C/T plus -31T/C). But one interesting result found was that the seronegative healthy group (without any gastric problem) had 33.3% gene polymorphism at -511C/T site. A study performed in Pakistan by Raza et al.^18^ described that IL-1B gene polymorphism at two sites (-511 and IRN) contributes a high risk for GC in patients carrying *H. pylori* infection and suggested that a low frequency polymorphism in healthy group may increase the susceptibility for *H. pylori* infection. In the Russian populations, a significant association was found among *H. pylori* infection and IL-1B polymorphism.^19^ In contrast, a study showed that single nucleotide polymorphism in IL-1B promoter site -511 was not linked with gastric disease while only IL-1B-31 position polymorphism was only linked with gastric disease risk in Caucasian population.^20^ Another study conducted for polymorphism in IL-1B at -511, -31 and IL-18 reported that IL-1B-511 polymorphism was responsible for causing the increase risk GC while other two positions were not significantly correlated for development of GC.^21^ In present study, IL-1B gene polymorphism was higher in gastric diseased groups (with or without *H. pylori* infection) as compared to seronegative healthy group.

In this study, we evaluated the relevance of sequence context of IL-1B gene polymorphs with haplotypic frequency predominance in the selected population. Three main haplotype variables (CC, TC and TT) with various rates were found in the promoter/enhancer sites of IL-IB (-511CT and -31TC). The frequencies of C/T haplotypes were identified by annotating the genotyping sequence results in software Snapgene viewer (version 4.2.1). In this particular study, for IL-1B-511T/C polymorphism, CC and TT haplotype frequencies were in 16.7%, while CT was found in 33.3% of healthy individuals. However, in the *H. pylori* seropositive patients with gastric diseases, risk was increased 20% for IL-1B-511 CC haplotype as compared to *H. pylori* seronegative carrying 14.3% CT haplotype. IL-1B-31 CC haplotype was observed higher 28.6% in seronegative gastric diseased group followed by 20% in *H. pylori* seropositive gastric diseased cases. While haplotypes TT and TC (16.7%) were found predominant in healthy individuals. Combined polymorphism of IL-1B-511 plus IL-1B-31 genotypes results showed that TT (28.6%) was high in *H. pylori* seronegative gastric diseased patients while in *H. pylori* seropositive patients, haplotype CC (20%) was observed. In China, a study conducted by Hong et al.^22^ described that interleukin IB-511TT or T allelic variants had a link for gastric carcinogenesis. Another study conducted in Germany, showed polymorphism of IL-1B gene was without any involvement of carcinogenesis.^23^ A comparative study investigated the haplotype frequencies (CC, TC and TT) in four Asian populations with gastric diseases and found Japan and Thailand population at higher risk of *H. pylori* infection as compared with China and Vietnamese population.^24^ In the present study, a potential association of allelic variants of IL-1B gene with gastritis was found. Study polymorphism results showed predominance of CC and TC was found in gastric diseased population of district Faisalabad. While a study conducted in Korea, reported a higher frequency of TT haplotype in -511 and -1RN positions polymorphism with an association of gastric diseases.^25^

## CONCLUSIONS

The present study showed only a higher expression of IL-1B-31 polymorphism in *H. pylori* infected gastric diseased patients as compared to IL-1B-511 polymorphism. So a partial involvement of studied gene contribution was observed. Both IL-1B-511 and 31 positions were not equally responded for gastric diseases development. The patients with age 41-60 years were found at higher risk of *H. pylori* infection. Although the studied gastric diseased patients were frequently found positive for *H. pylori*, it is not only the risk for gastric complications. The *H. pylori* positive population having IL-IB polymorphism was presented with gastritis, EFV and PU that increase the risk of adenocarcinoma. It is suggested that upon diagnosis with *H. pylori* infection, the in time therapy be considered as compulsory to the patients. Moreover, IL-1B-511 and IL-1B-31 CT polymorphism that was also observed in healthy individuals may increase the risk of getting *H. pylori* infection.

### Author`s Contribution

**FK and SUR** did study design and conceived study concepts.

**FK** did data acquisition, quality control of data, data analysis, interpretation and manuscript writing.

**FK and SUR** did data analysis, interpretation and statistical analysis.

**SUR, MSM and TZ** did manuscript editing and review.

All authors have approved the final version to be published.

## References

[1] Das D, Abbas M, Akabar M, Nepal A, Islam MA, Ayuba A (2016). A clinical review on the pathology and management “Helicobacter pylori”infection. Int J Adv Res Biol Sci.

[2] Loesnihari R (2018). Detection of H. Pylori infection on dyspepsia patients with IgA H. Pylori antibody. InIOP Conference Series:Earth Env Sci.

[3] Sadeghi RN, Damavand B, Vahedi M, Mohebbi SR, Zojazi H, Molaei M (2013). Detection of p53 common intron polymorphisms in patients with gastritis lesions from Iran. Asian Pac J Cancer Prev.

[4] Yang XJ, Si RH, Liang YH, Ma BQ, Jiang ZB, Wang B (2016). Mir-30d increases intracellular survival of Helicobacter pylori through inhibition of autophagy pathway. World J Gastroenterol.

[5] Venerito M, Selgrad M, Malfertheiner P (2013). Helicobacter pylori:gastric cancer and extragastric malignancies—clinical aspects. Helicobacter.

[6] Zhao Y, Wang JW, Tanaka T, Hosono A, Ando R, Tokudome S (2013). Association between TNF-αand IL-1βgenotypes vs Helicobacter pylori infection in Indonesia. World J Gastroenterol.

[7] Abbas Z, Moatter T (2003). Interleukin (IL) 1b and IL-10 gene polymorphism in chronic hepatitis C patients with normal or elevated alanine aminotransferase levels. J Pak Med Assocc.

[8] Ramis IB, Vianna JS, Gonçalves CV, von Groll A, Dellagostin OA, da Silva PE (2017). Polymorphisms of the IL-6, IL-8 and IL-10 genes and the risk of gastric pathology in patients infected with Helicobacter pylori. J Microbiol Immu Infect.

[9] Chang YW, Jang JY, Kim NH, Lee JW, Lee HJ, Jung WW (2005). Interleukin-1B(IL-1B) polymorphisms and gastric mucosal levels of IL-1βcytokine in Korean patients with gastric cancer. Int J Cancer.

[10] Sun X, Cai H, Li Z, Li S, Yin W, Dong G (2017). Association between IL-1βpolymorphisms and gastritis risk:A meta-analysis. Medicine.

[11] Cheng HH, Chang CS, Wang HJ, Wang WC (2010). Interleukin?1βand?10 polymorphisms influence erosive reflux esophagitis and gastritis in Taiwanese patients. J Gastroenterol Hepatol.

[12] Ding Q, Fan B, Fan Z, Ding L, Li F, Tu W (2013). Interleukin-10-819C>T polymorphism contributed to cancer risk:evidence from 29 studies. Cytokine.

[13] de Brito BB, da Silva FA, de Melo FF (2018). Role of polymorphisms in genes that encode cytokines and Helicobacter pylori virulence factors in gastric carcinogenesis. World J Clinical Oncol.

[14] Ma J, Wu D, Hu X, Li J, Cao M, Dong W (2017). Associations between cytokine gene polymorphisms and susceptibility to Helicobacter pylori infection and Helicobacter pylori related gastric cancer, peptic ulcer disease:A meta-analysis. PLoS One.

[15] Parsonnet J, Friedman GD, Vandersteen DP, Chang Y, Vogelman JH, Orentreich N (1991). Helicobacter pylori infection and the risk of gastric carcinoma. N Engl J Med.

[16] Terry CE, McGinnis LM, Madiga KC, Cao P, Cover TL (2005). Genomic comparison of cag pathogenicity island (PAI)-positive and-negative Helicobacter pylori strains:identification of novel markers for cag PAI-positive strains. Infect Immun.

[17] Tongtawee T, Kaewpitoon S, Kaewpitoon N, Dechsukhum C, Leeanansaksiri W, Loyd RA (2016). Characteristics and risk factors of Helicobacter pylori associated gastritis:a prospective cross-sectional study in Northeast Thailand. Gastroenterol Res Pract.

[18] Raza Y, Khan A, Khan AI, Khan S, Akhter S, Mubarak M (2017). Combination of interleukin 1 polymorphism and Helicobacter pylori infection:an increased risk of gastric cancer in Pakistani population. Pathol Oncol Res.

[19] Kulmambetova GN, Imanbekova MK, Logvinenko AA, Sukashev AT, Filipenko ML, Ramanculov EM (2014). Association of cytokine gene polymorphisms with gastritis in a Kazakh population. Asian Pac J Cancer Prev.

[20] Murphy G, Thornton J, McManus R, Swan N, Ryan B, O'Morain CA (2009). Association of gastric disease with polymorphisms in the inflammatory related genes IL-1B, IL-1RN, IL-10, TNF and TLR4. Eur J Gastroenterol Hepatol.

[21] Ma J, Wu D, Hu X, Li J, Cao M, Dong W (2017). Associations between cytokine gene polymorphisms and susceptibility to Helicobacter pylori infection and Helicobacter pylori related gastric cancer, peptic ulcer disease:A meta-analysis. PLoS One.

[22] Hong JB, Zuo W, Wang AJ, Lu NH (2016). Helicobacter pylori infection synergistic with IL-1βgene polymorphisms potentially contributes to the carcinogenesis of gastric cancer. Int J Med Sci.

[23] Wex T, Leodolter A, Bornschein J, Kuester D, Kaehne T, Kropf S (2010). Interleukin 1 beta (IL1B) gene polymorphisms are not associated with gastric carcinogenesis in Germany. Anticancer Res.

[24] Matsukura N, Yamada S, Kato S, Tomtitchong P, Tajiri T, Miki M (2003). Genetic difference in interleukin-1 beta polymorphisms among four Asian populations:an analysis of the Asian paradox between H. pylori infection and gastric cancer incidence. J Exp Clin Cancer Res.

[25] Kim JJ, Kim N, Hwang S, Kim JY, Kim JY, Choi YJ (2013). Relationship of interleukin-1βlevels and gastroesophageal reflux disease in Korea. J Gastroenterol Hepatol.

